# Draft Genome Sequence of the Type Strain Streptomyces armeniacus ATCC 15676

**DOI:** 10.1128/MRA.01107-18

**Published:** 2018-09-20

**Authors:** Puneet Labana, Jessica T. Gosse, Christopher N. Boddy

**Affiliations:** a Department of Chemistry and Biomolecular Sciences, University of Ottawa, Ottawa, ON, Canada; Queens College

## Abstract

The Streptomyces genus represents a prolific and significant source for secondary metabolite discovery. Here, we report a *de novo* assembly and draft genome sequence for the type strain Streptomyces armeniacus ATCC 15676.

## ANNOUNCEMENT

Streptomyces spp. are known producers of a diverse array of bioactive natural products, including antibiotics, antifungals, and anticancer agents (1). As such, considerable effort has been spent examining and sequencing environmental isolates to characterize their ability to produce these important molecules (2, 3). However, to complement this effort, it is essential to also adequately characterize Streptomyces type strains, as they establish the points of reference that other Streptomyces strains are compared with (4). Here, we sequenced the complete genome of Streptomyces armeniacus ATCC 15676 (NBRC 12555, BCRC 16847, RIA 807, 26A-32, KCC A-0070, NCIB 10179) type strain, isolated from soil of Armenian origin (5, 6), and assembled a high-quality draft genome sequence.

The Streptomyces strain was revived per ATCC guidelines and cultivated in ISP-2 medium at 26°C for 1 week (7, 8). The genomic DNA (gDNA) was then extracted using a Wizard genomic DNA purification kit (Promega). Purified gDNA was sent to Dalhousie University (Halifax, NS, Canada) for sequencing at the Centre for Comparative Genomics and Evolutionary Bioinformatics (CGEB). Sequencing results were obtained using an Illumina MiSeq DNA sequencer. The library was prepared with a Nextera DNA sample preparation kit (Illumina), following the manufacturer’s instructions, and sequenced using a MiSeq 600-cycle v3 reagent kit (Illumina).

*De novo* assembly of paired-end files was performed using a SPAdes protocol in the PATRIC genome assembly service (version 3.5.18) (9). Quality control of reads was set to a minimum contig coverage of 5× and a minimum contig length of 300 bp. The resulting draft genome sequence consists of 12 contigs with a total genome size of 8,073,679 bp and a G+C content of 72.4%. The average read length is 3,354,904 bp with an *N*_50_ value of 2,357,927 and coverage of 65×.

Subsequently, an automated annotation was performed with Rapid Annotations using Subsystem Technology (RAST) version 2.0 (10). A total of 7,080 coding sequences and 68 RNAs were predicted. Biosynthetic gene clusters (BGCs) were identified through antiSMASH version 4.2.0 (11). In total, 36 BGCs related to multiple different secondary metabolites were predicted. Mixed pathway products, along with the type 1 polyketide synthases (PKSs) and nonribosomal peptide synthetases (NRPSs) were most abundant, with 10, 7, and 6 BGCs, respectively. Following were 4 BGCs encoding ribosomal natural products and 4 BGCs encoding terpene pathways. A single prediction each for a siderophore, type 2 PKS, and type 3 PKS BGC were also identified. It is noteworthy that while this type strain is not documented to produce any secondary metabolites, it clearly possesses significant genetic potential, which further validates the utility of type strain sequencing.

### Data availability.

This whole-genome sequence has been deposited at DDBJ/ENA/GenBank under the accession number CP031320. The version described in this paper is the first version.
